# Cardiac Chagas Disease: MMPs, TIMPs, Galectins, and TGF-*β* as Tissue Remodelling Players

**DOI:** 10.1155/2019/3632906

**Published:** 2019-11-25

**Authors:** Arthur Wilson Florencio da Costa, Jose Rodrigues do Carmo Neto, Yarlla Loyane Lira Braga, Beatriz Aquino Silva, Amanda Borges Lamounier, Bárbara Oliveira Silva, Marlene Antônia dos Reis, Flávia Aparecida de Oliveira, Mara Rúbia Nunes Celes, Juliana Reis Machado

**Affiliations:** ^1^Institute of Tropical Pathology and Public Health, Federal University of Goiás, 74605-450 Goiânia, GO, Brazil; ^2^Faculty of Medicine, Federal University of Goiás, 74605-050 Goiânia, GO, Brazil; ^3^Department of General Pathology, Federal University of Triângulo Mineiro, 38025-180 Uberaba, MG, Brazil

## Abstract

A century after the discovery of Chagas disease, studies are still needed to establish the complex pathophysiology of this disease. However, it is known that several proteins and molecules are related to the establishment of this disease, its evolution, and the appearance of its different clinical forms. Metalloproteinases and their tissue inhibitors, galectins, and TGF-*β* are involved in the process of infection and consequently the development of myocarditis, tissue remodeling, and fibrosis upon infection with *Trypanosoma cruzi*. Thus, considering that the heart is one of the main target organs in Chagas disease, knowledge regarding the mechanisms of action of these molecules is essential to understand how they interact and trigger local and systemic reactions and, consequently, determine whether they contribute to the development of Chagas' heart disease. In this sense, it is believed that the inflammatory microenvironment caused by the infection alters the expression of these proteins favoring progression of the host-parasite cycle and thereby stimulating cardiac tissue remodeling mechanisms and fibrosis. The aim of this review was to gather information on metalloproteinases and their tissue inhibitors, galectins, and TGF-*β* and discuss how these molecules and their different interrelationships contribute to the development of Chagas' heart disease.

## 1. Introduction

Chagas disease is caused by infection of the flagellated protozoan *Trypanosoma cruzi* (*T. cruzi*), a parasite native to Latin America, which is an endemic region of the disease. Currently, about 6 to 7 million people worldwide are infected by this parasite, and in Brazil, this number reaches 1 million. Additionally, approximately 20 million individuals live in areas which are at a risk for this disease [1].

This disease is usually manifested asymptomatically and may evolve with different clinical manifestations characterizing its different forms, which are as follows: cardiac, digestive, and cardiodigestive. After infection, the individual goes through an acute phase, in which nonspecific symptoms are usually presented [2]. The disease progresses for 20 years or more, wherein the symptoms become chronic and more severe, and, when left untreated, leads to death of the individual [3, 4].

Cardiac involvement results in the development of heart disease, which is generally the most severe form of Chagas disease affecting about 20-30% of infected individuals [5]. Chagasic cardiomyopathy is a complex process resulting in the destruction of cardiac cells, cellular hypertrophy, alteration of electrical signals, dilatation of the cardiac chambers, proliferation of cardiac fibroblasts, and tissue matrix remodeling [2, 3, 6]. According to the World Health Organization (WHO), there are about 50,000 deaths per year associated with chagasic cardiomyopathy and related to sudden cardiac death (60%), heart failure (25%), and strokes (15%) [7–10].

In the acute phase of infection, cardiomyopathy is characterized by an intense infiltration of inflammatory, mono, and polymorphonuclear cells, which release inflammatory cytokines and chemokines favoring continuous cell recruitment. In the chronic phase, the profile of infiltrated cells is predominantly mononuclear [11] and cardiac damage is mainly associated with inflammation caused by parasitism in the cardiomyocytes, tissue aggression resulting from the immune response [12], and high intensity of tissue remodeling, which is a product of collagen fiber deposition [13].

Additionally, these phenomena of parasitism, immune response, and tissue matrix remodeling are directly dependent on the interaction of numerous proteins, the expressions of which have been altered due to infection. Matrix metalloproteinases (MMPs) [14, 15], specific tissue metalloproteinase inhibitors (TIMPs) [16, 17], galectins (Gals) [17–20], and the transforming growth factor-beta (TGF-*β*) [21] are examples of molecules that have been studied in the context of Chagas disease in both experimental and human models.

MMPs are molecules directly involved with cardiac remodeling because they occur constitutively and are responsible for the degradation of extracellular matrix components [22], and TIMPs, at appropriate levels, are responsible for regulating the activity of the MMPs in the tissues. Infection with *T. cruzi* results in a proinflammatory environment causing an increased expression of MMPs 2 and 9. In this context, the high expression of MMPs inhibitors, TIMP-1 and TIMP-2, has been associated with the severity of the fibrotic process in experimental chagasic cardiomyopathy. In the process of cardiac tissue remodeling, galectins can activate collagen-producing cells as well, stimulating the production of proinflammatory cytokines such as TGF-*β*. This growth factor, in turn, acts on the tissue repair process and cardiac matrix remodeling.

Supposedly, this complex network of interactions involving these molecules depends directly on the inflammatory microenvironment and protozoan biological characteristics associated with the Chagas disease. This review proposes a complete understanding of the correlation of these proteins, as significant molecules in the cardiac tissue that help in understanding the pathophysiology of Chagas' heart disease at the expense of tissue remodeling.

## 2. The Role of Matrix Metalloproteinases in the Pathophysiology of Chagas Disease

MMPs are regulatory proteins involved in important physiological processes such as embryogenesis, angiogenesis, and tissue remodeling and may be inappropriately activated in various pathological processes, such as Chagas' heart disease, resulting in cell dysfunction and consequent tissue destruction [22].

MMPs form a group of enzymes (endopeptidases) comprising collagenases, gelatinases, stromelysins, matrilysins, membrane-like MMPs (MT-MMPs), and other MMPs responsible for degradation of the extracellular matrix (EM) and basement membrane components [23, 24]. Under physiological conditions in the myocardium, these enzymes may be inactive and inhibited by a group of proteins known as TIMPs. It has been demonstrated that these enzymes are expressed in different parasitic diseases, including Chagas disease. In this context, changes in the interactions between these molecules appear to be correlated with the changes in the myocardial tissue matrix observed in Chagas disease, and studies suggest that this imbalance affects both the geometry and overall cardiac function [23, 24].

According to Geurts et al. [25], the production of MMPs may also occur directly by the parasite, and the process of fibrogenesis is favored by uncontrolled levels and activity of MMPs and, consequently, results in the development of cardiomyopathy suggesting that MMPs may be an interesting therapeutic target. Among the MMPs, the following deserve special mention with respect to Chagas disease: MMP-2, as it is the most abundant and naturally expressed molecule in all cells, including cardiomyocytes, and plays important roles in homeostasis and the constant remodeling of matrix, and MMP-9, which is a cytokine-induced proteinase produced by different cell types [26]. The transcription and activation of these MMPs occur due to an increased expression of several cytokines such IL-6, IL-8, and TGF-*β*, which are induced in the inflammatory state. This phenomenon is observed in the acute phase of Chagas disease at the expense of strong seropositivity for *T. cruzi* [27].

Interestingly, it has already been shown by Pérez et al. [28] and Savino [29] that infectious agents, such as *T. cruzi*, in the endocrine system may contribute to the recruitment of inflammatory cells and the release of proinflammatory cytokines that contribute to the production extracellular matrix stimulating and maintaining more inflammatory processes.

Bautista-López et al. [22] demonstrated by densitometry analysis using blood plasma samples that a significant increase in the levels of MMP-2 and MMP-9 occurs in patients infected with *T. cruzi*. They also demonstrated a significant increase in the MPP activity when compared to the electrocardiogram of patients with cardiomyopathy and the control groups. They found that abnormal cardiac relaxation can be positively correlated with high levels of MMP-2 in patients with cardiac dilation occurring due to cardiomyopathy and then suggested that the MMPs 2 and 9 may act as potential biomarkers that can be used to identify not only the advent of cardiomyopathy but also its occurrence and progression in individuals infected with *T. cruzi*.

The hearts of wild-type mice, in which MMP-2 was overexpressed, were evaluated by Bergman et al. [30], who observed that significant cardiac damage such as cardiac dilation and thinning of the ventricular walls was associated with the high levels of expression of this molecule. Still, in this sense, other authors have reinforced the idea that MMPs 2 and 9 are involved in the pathogenesis of Chagas disease as they are able to degrade the components of the cellular matrix components in the heart, which contributes, at least in part, to cardiac tissue remodeling [27].

This cardiac remodeling is dependent on the type of MMPs produced. Hence, the action of MMP-9 on the myocardium may be by exacerbating the remodeling and contributing to the evolution of the heart shape development of the disease while, on the other hand, increasing the levels of MMP-2. Interestingly, this microenvironment results in a more discreet cardiac remodeling, contributing in some way such that the host remains in the undetermined phase of the disease. In this sense, Gutierrez et al. [31] have demonstrated that a significant regression of the cardiac inflammatory process and improved survival rates were observed in animals treated with the MMP-2 and MMP-9 inhibitors. The authors have further reinforced the role of MMPs in chagasic myocarditis induced by *T. cruzi* as well as suggesting a possible therapeutic target for heart disease caused by Chagas disease, which, however, needs further study.

It is undeniable that MMPs 2 and 9 are not only involved in stimulating tissue damage but also contribute to the exacerbation of the inflammatory response by activating various cytokines and chemokines and by costimulating tissue repair by depositing EM proteins [15]. It is clear these MMPs play a significant role in the pathogenesis of Chagas' heart disease, sometimes by stimulating the inflammatory process and cardiac remodeling and otherwise by curiously regulating these processes negatively. The findings of this review support the real need for further studies evaluating the role of MMPs as well as their inhibitors (TIMPs) in Chagas disease.

## 3. Tissue Inhibitors of MMPs in Chagas' Heart Disease

TIMPs, as regulators of MMPs, comprise a group of four molecules that bind with high affinity to the active MMPs and ultimately result in their proteolytic inactivation. These molecules are therefore known as the key regulators of MMPs [32]. This interaction between the TIMPs and MMPs occurs specifically and is of particular interest in the context of Chagas disease. TIMPs 2, 3, and 4 and TIMPs 1 and 3 inhibit MMP-2 and MMP-9, respectively [33].

According to Brew and Nagase [16] an imbalance in the production of these active enzymes and/or their inhibition may result in the development of diseases associated with extracellular matrix rearrangement, exacerbation of the inflammatory process, growth, and cell migration, which are phenomena frequently observed in Chagas disease. These authors have also demonstrated that the biological effects of TIMPs, such as the influence on cell differentiation and migration, synaptic plasticity, and antiangiogenic and anti-proapoptotic activities, can be MMP-independent.

Among the different biological activities of TIMPs, type 1 is an important molecule involved in the regulation of cardiac remodeling, as demonstrated by Roten et al. [34], who evaluated that important changes associated with impaired cardiac function took place in the left ventricles of mice that did not express this enzyme. TIMP-1 acts by promoting fibroblast growth by activating the mitogen-activated protein kinase (MAP) leading to increased levels of Ras-GTP, which in turn results in increased levels of collagen favoring the occurrence of fibrosis [16].

However, it has been observed that the overexpression of TIMP-1 after gene therapy does not appear to be an effective tool in preventing cardiac remodeling. Gutierrez et al. [31] reported that, in *T. cruzi* infection, the expression of TIMP-1 was associated with an increased induction of collagen synthesis, thus favoring cardiac fibrosis. The overexpression of these TIMPs may even contribute to the pathogenesis of the chronic phase of the disease, in which an exacerbated fibrotic response of the cardiac form is observed.

On the other hand, some authors evaluating the knockout of TIMP-3 mice in different organs had observed an increase in the lung airspace and occurrence of apoptotic cell death during mammary gland involution [35]. According to these authors, both phenomena could have resulted from defects in the inhibition of MMPs, which reinforces the importance of the biological role of TIMPs, which can contribute to matrix degradation by preventing the inactivation of MMPs. This imbalance of TIMPs and MMPs involved in the process of matrix degradation was also observed in the heart, which resulted in cardiomyopathy [36]. Interestingly, Geurts et al. [24] have shown that uncontrolled activities of MMPs as well as the overregulation of MTPs may favor fibrogenesis and, consequently, may result in the development of cardiomyopathy.

In an experimental study by Gutierrez et al. [31], mice with induced Chagas' heart disease showed increased levels of both TIMP-1 and TIMP-3 but a direct relationship with the TIMP-2 levels was not observed. This corroborates the findings of Bergman et al. [30], who demonstrated that the overexpression of MMP-2 is associated with lower cardiac tissue remodeling and is able to reduce the progression of Chagas' heart disease.

Therefore, although Gutierrez et al. [31] revealed that the TIMPs had acted against the counterregulation of MMPs, this response may be considered to be insufficient in preventing myocardial damage. TIMPs can also modulate other critical signaling pathways independent of MMP inhibition, thus suggesting that an understanding of the mechanisms of action of these enzymes should be explored for a better understanding of the pathogenesis of Chagas disease [37] as well as its potential biomarkers.

There is no doubt about the importance of the role of TIMPs in the regulation of the MMPs involved in the process of tissue matrix remodeling, both physiologically and pathologically, and their involvement can be detected during cardiac remodeling in Chagas' heart disease.

## 4. The Dualistic Action of Galectins in Chagasic Cardiomyopathy

Galectins are glycan-binding proteins similar to the MMPs and are widely expressed in various cell types. They are mainly present in the cytoplasm but may also be found in the nucleus, cell surface, and extracellular environment [38, 39]. Fifteen members of the galectin family have been described in vertebrates [40], two of which have been identified as EC-specific proteins, which are as follows: galectins 1 (Gal-1) and 3 (Gal-3).

These proteins have a range of effects associated with the inflammatory processes [20] and are related and expressed under the conditions of fibrogenesis and myocardial failure [17]. Their functions remain uncertain, and studies have contradictively demonstrated their positive and negative effects on the pathophysiology of Chagas disease [18, 41].

High levels of Gal-1 [42] have been reported in the hearts of patients with Chagas' heart disease although cells such as the B lymphocytes [43] produce high levels of Gal-1 when infected with *T. cruzi*. This lectin is believed to be released into the extracellular medium only after cell lysis of the myocardiocytes mediated by the trypomastigote forms [18].

Poncini et al. [41] demonstrated a negative role of Gal-1 in Chagas disease after infection and during immune response modulation, which contributed to the process of cellular infection. The authors demonstrated that dendritic cells (DCs), which play a crucial role in initiating the immune response, interact with galectins during the acute phase of infection and promote tolerance of the immune system to the parasite. This occurs by stimulating the differentiation of tolerogenic dendritic cells and regulatory T cells, which favors the evolution of the disease. According to the same authors, DCs play a different role, in which they prevent the induction of an inflammatory response. They promote T cell anergy and stimulate regulatory T cells, which suppresses the inflammatory response and, consequently, tissue damage.

Benatar et al. [18] demonstrated that, in a HL-1 myocardiocyte lineage, the presence of galectin decreases *T. cruzi* cell infection, as well as phosphatidylserine exposure, which is important for the induction of apoptosis. These authors demonstrated that if the extent of parasitemia in Lgals1^−/−^ animals infected with the Tulahuén strain was higher, the survival rates were lower compared to those of the wild animals.

Additionally, a gene study has suggested that infection with *T. cruzi* increases the expression of the LGASL3 gene responsible for coding another galectin, Gal-3 [42]. Gal-3 plays a key role in the adhesion of the trypomastigote forms to the host cells during the initial process of infection. In this process, Gal-3 binds to parasite-specific mucins and interacts with laminins [43] increasing their adhesion to the EC components [43, 44], favoring the accumulation of tissue parasites and causing cardiomyocyte infection [44].

Pineda et al. [19] demonstrated that the affinity with which this lectin binds to the intracellular amastigotes is greater than that with the trypomastigote form, in which binding to the galactosides occurs on the surface of the parasite. Using immunofluorescence, it was observed that the amastigote forms are coated with a pool of cytosol galectins. Based on these observations, the authors have suggested that Gal-3 increases the extent of *T. cruzi* cell infection. This would occur as the released cell amastigotes would increase and alter the pool of galectins favoring the adhesion and invasion of new cells more rapidly. The authors also suggest that other mechanisms such as the rearrangement of the structures of the galectin receptor would allow the amastigotes to survive in the extracellular environment. Alternatively, the labeling of extracellular amastigotes in order to enable the macrophages to detect and phagocyte the protozoan would also contribute to the infection.

Contrarily, under physiological conditions, Gal-3 is involved in the binding of CD with T cells after activation of the L-selectins. In vivo and in vitro studies have shown that *T. cruzi* infection increases the concentration of Gal-3 in the acute phase as well as in their CD binding sites. Additionally, the migration capacity of CD is found to be impaired. Because these cells promote antigen presentation, it may characterize a negative immunomodulation mechanism promoted by *T. cruzi* [42].

In addition to CD, the expression of this lectin is also higher in the B lymphocytes upon infection, and this increase promotes a negative effect on the activity of interleukin 4. This results in a lack of stimulation and inhibition of plasma cell differentiation, subsequent decrease in the antibody production, and parasite clearance favoring the evolution of the disease [45].

Some studies have shown that in the evolution of Chagas' heart disease, which results in intense cardiac remodeling, Gal-3 also participates in the process of collagen production. Gal-3 induces the proliferation of cardiac fibroblasts converting them to myofibroblasts and thereby stimulating TGF-*β* synthesis [17, 39, 46]. By mediating TGF-*β* production, Gal-3 could be involved in the activation of a profibrotic pathway known as TGF-*β*1/*α*-SMA/Col-1, as demonstrated by Henderson et al. [46] in a model of atrial fibrosis. This pathway results in the production of TGF-*β*1, procollagen, and *α*-SMA, which are important components involved in the differentiation of fibroblasts into myofibroblasts [47], which may be important sources of Gal-3 in pathological conditions such as the Chagas disease [39, 48].

The expression of myocardial lectin in mice infected with *T. cruzi* is high [42, 45]. In addition to cardiac fibrosis, other studies have shown that Gal-3 is also involved in the development of fibrosis in other organs [46–49]. This suggests that the expression of Gal-3 would then be directly related to cardiac remodeling.

Contrarily, when infected with the Colombian strain and treated with N-Lac, a Gal-3 blocker, mild fibrosis and myocardial inflammatory cell migration were observed in the mice [50]. In this sense, Ferrer et al. [51] observed that experimental infection with *T. cruzi* results in an increased expression of profibrotic genes, which include the following: Col-1, *α*-SMA, and Gal-3 in regions of fibrosis and inflammation in the myocardium. Pineda et al. [19] have reinforced these findings by revealing that a reduction in the inflammatory infiltrate and mild myocardial fibrosis was observed in the Lgals1^−/−^ mice. The same observations were noted when using G-CSF, a Gal-3 modulator [52].

Thus, finding alternatives that interfere with the expression of Gal-3 in patients with the indeterminate form of Chagas disease, who are naturally at an imminent risk of developing Chagas' heart disease, seems promising [52]. According to Pineda et al. [19], a deficient Gal-3 phenotype is compatible with an anti-inflammatory profile at the expense of promoting deregulation in the Toll-like receptor expression. These authors have demonstrated that, during *T. cruzi* infection, these effects observed in the deficient Gal-3 phenotype would increase blood parasitism but not cardiac parasitism in the antigen-presenting cells and further reduce cytokine production, inflammation, and myocardial fibrosis.

Therefore, Gal-3 is involved in the pathophysiology of the cardiac form of Chagas disease. Thus, studies on these lectins in chagasic cardiomyopathy models may result in the identification of new therapeutic targets leading to better prognosis.

## 5. TGF-*β*, a Marker of the Progression of Chagas' Heart Disease

TGF-*β* is a cytokine belonging to the group of the “TGF-*β* superfamily” and has three isoforms, TGF-*β*1, 2, and 3. This cytokine participates in immune response modulation and inflammation and is involved in cellular growth mechanisms, differentiation, and cell death [53]. Additionally, it plays an important role in collagen synthesis and is considered as one of the main profibrotic factors stimulating the phenotypic transition from the fibroblast to its effector fibrotic form, the myofibroblast [54].

Under normal conditions, this cytokine stimulates collagen production and inhibits MMP activity through the synthesis of protease inhibitors [55, 56]. During cardiac injury, it in turn promotes tissue remodeling and repair [56]. Some studies have shown that TGF-*β* plasma and cardiac elevated levels are associated with some type of cardiac dysfunction [57–60] such as Chagas' heart disease [55]. A 10- to 20-fold increase in serum TGF-*β*1 levels has been observed in CD patients compared to uninfected healthy subjects. Elevated serum levels of this cytokine have been associated with more severe forms of heart disease in chronic patients [55, 61].

TGF-*β* is produced in the individuals in the acute and chronic stages of the Chagas disease. Thus, it participates in different processes influencing progression of the disease. For example, it has been shown that cell invasion by the Silvio and Tulahuén strains is dependent on TGF-*β* signaling, since infection was extremely inefficient in MvlLu cells with T*β*RI and T*β*RII receptor defects [62]. Using the strain Y, Waghabi and colleagues [63] demonstrated that in vitro treatment of cardiomyocytes with anti-TGF-*β* antibody slowed the process of cell invasion. Other authors suggest that this dependence was due in part to the SMAD pathway [64], since the binding of TGF-*β* to its receptors stimulates phosphorylation of intracellular proteins such as Smad2/3 [65].

TGF-*β* appears to favor infection as it is released from cells in its inactive form due to binding with the latency-associated protein (LAP) [66, 67]. In order to enable binding of this cytokine to its receptor, it is necessary to break the interaction of LAP with TGF-*β*, which is catalyzed by numerous agents including integrins [66], thrombospondin [68], and proteases (plasmin, MMP-2, and MMP-9) [68, 69], which would activate the latent TGF-*β*.

This latent or inactive form of TGF-*β* can be activated by both the amastigote and trypomastigote forms of the strains Y and Dm28, which favor the parasite cycle and facilitate host cell infection [63]. Similar mechanisms have also been observed in infections by other protozoa such as *Leishmania* [70, 71] and *Plasmodium* [72].

This activation may also be directly related to the exacerbated production of EC components [73], as demonstrated in a cardiac biopsy analysis of patients with Chagas disease. In these cases, an increase in the fibrosis associated with high levels of TGF-*β* was observed by perivascular fibronectin and strong tissue phosphorylated SMAD-2 nuclear labeling [55].

Nevertheless, the activation of latent TGF-*β* dependent on *T. cruzi* is still a poorly studied mechanism and is believed to occur mainly via the action of cruzipain, a peptidase expressed at all stages of the parasitic infection [73]. Waghabi et al. [63] suggested that TGF-*β* is captured and accumulated within the parasite mainly in the amastigote phase, suggesting that this cytokine plays an important role during the multiplication and differentiation period. The authors have suggested that this accumulation occurs through the “flagellar pocket,” which is capable of encompassing the TGF-*β*-rich vesicles from the host endoplasmic reticulum during the infection process.

Some authors believe that after the period of infection and during the development of the inflammatory process and immune response, this cytokine increases the replication of *T. cruzi* at the expense of inhibiting the microbicidal effects on macrophages induced by IFN-*γ* [74–76]. This would contribute to the evolution of the disease and cardiac dysfunction. In an in situ evaluation of the heart of patients with chronic Chagas disease, no significant relationship was found between the TGF-*β* expression and cardiac dysfunction [77]. However, the role of TGF-*β* as a profibrotic factor has been reported in other pathological processes: diabetic nephropathy in in vitro models and experimental models [78, 79], chronic intestinal inflammation [80], and rheumatoid arthritis [81, 82].

In this case, a series of molecules are being developed in an attempt to intervene in the TGF-*β* pathway, which are as follows: oligonucleotides, aiming at blocking the synthesis of TGF-*β*1 and TGF-*β*2 ligands; monoclonal antibodies capable of neutralizing the different TGF-*β* isoforms; synthetic peptides containing TGF-*β* receptor domains capable of sequestering free TGF-*β* from the extracellular medium; and small chemical compounds that block signal transduction of the TGF-*β* pathway [73].

These pharmacological inhibitors of TGF-*β* receptors such as SB-431542 (type I) and GW-788388 (type I and type II) have been tested in a murine experimental model for Chagas' heart disease. In an experimental evaluation of the acute phase with the strain Y, the mice were treated intraperitoneally with SB-431542 three days after infection. After treatment, a reduction in the levels of parasitemia, mortality rates, inflammatory infiltrate, parasitic load, and electrical conduction in the heart of the infected and treated animals [83] was observed. A decrease in the number of intracellular amastigotes and the release of trypomastigotes were observed upon in vitro evaluation of SB-431542-treated cardiomyocytes infected with the strain Y and Dm28c, which reinforces the role of this cytokine in the process of cellular infection and differentiation [76].

In an analysis performed on SB-431542 and *T. cruzi*, it was observed that inhibition of the TGF-*β* pathway resulted in a decrease in parasitic load and fibronectin expression in a three-dimensional model of cell culture “cardiac spheroids” that reproduced an infection similar to that observed in vivo after Y strain infection. As collagen production depends on the balance between its production and degradation, mainly mediated by MMPs and TIMPS activity, a decrease in MMP-2 and MMP-9 activity was observed in infected cells. Thus, after treatment, a significant increase in MMP-2 activity was observed, as well as a reduction in TIMP-1 activity in infected cells [84].

Mice with chronic Chagas' heart disease infected by the Colombian strain were treated orally with GW788388, an inhibitor of TGF-*β* type I and II receptor kinases. Although the treatment showed no effects on parasite load on the myocardium, the treatment was able to reverse cardiac fibrosis, showing lower levels of type 1 collagen and fibronectin when compared to untreated infected mice. The treatment was also able to improve cardiac function, and this may be associated with the high levels of MMP-9 and the low levels of TIMP-1, TIMP-2, and TIMP-4. In addition, the same authors demonstrated that treatment with GW788388 induced a lower influx of CD3^+^ cells into the heart of infected mice [85].

Thus, TGF-*β*, which was evaluated using different experimental models, presents itself as an important cytokine involved in the progression of Chagas' heart disease. When interacting with the trypomastigote and amastigote forms, this cytokine favors myocardiocyte infection and intracellular multiplication of the parasite. Thus, use of inhibitors of the TGF-*β* activation pathway may affect the parasite cycle and result in lower levels of parasitemia and cell infection. Thus, these therapies seem promising in reducing cardiac fibrosis and a better prognosis on the evolution of myocarditis.

## 6. Conclusions

**Figure 1:**
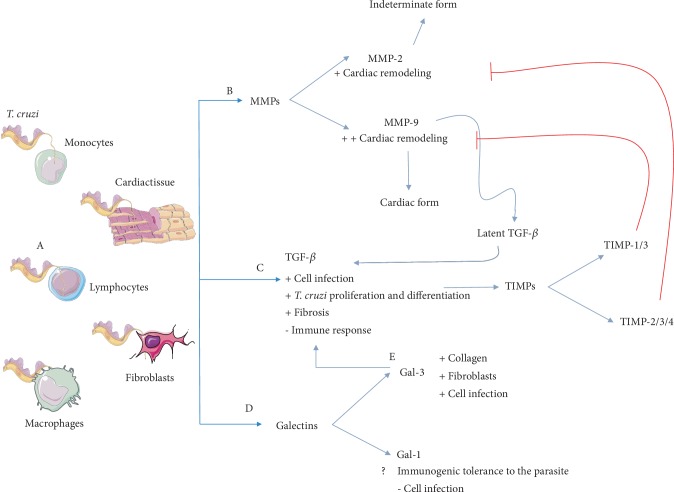
(A) Scheme representing the interrelation and regulation between metalloproteinases (MMPs), tissue inhibitors of metalloproteinases (TIMPs), galectins, and TGF-*β* in *Trypanosoma cruzi* infection. (B) MMPs act primarily on cardiac remodeling and activate latent TGF-*β*. (C) This cytokine supports the parasite intracellular cycle and stimulates fibrosis and cardiac damage. May also increase TIMP levels, favoring inhibition of MMPs. (D) While galectin-1 function is still controversial, this protein may be related to decreased protozoal infection and proinflammatory immune response. (E) Galectin-3 also stimulates cardiac remodeling and favors the parasite cycle via its own mechanism and also by stimulating TGF-*β* production. This figure is a derivative of “Servier Medical Art” by Servier, used under CCBy 3.0.

The inflammatory microenvironment generated by the Chagas disease results in an imbalance in the production, secretion, and activities of MMPs, TIMPs, galectins, and TGF-*β*. The interaction of these molecules (Figure 1), in different ways, directly or indirectly favors both, the infection and cardiac remodeling processes, consequently causing fibrosis that results in Chagas' heart disease.
